# The biosecurity benefits of genetic engineering attribution

**DOI:** 10.1038/s41467-020-19149-2

**Published:** 2020-12-08

**Authors:** Gregory Lewis, Jacob L. Jordan, David A. Relman, Gregory D. Koblentz, Jade Leung, Allan Dafoe, Cassidy Nelson, Gerald L. Epstein, Rebecca Katz, Michael Montague, Ethan C. Alley, Claire Marie Filone, Stephen Luby, George M. Church, Piers Millett, Kevin M. Esvelt, Elizabeth E. Cameron, Thomas V. Inglesby

**Affiliations:** 1 Future of Humanity Institute, Oxford University, Oxford, UK; 2 Alt. Technology Labs, Inc., Berkeley, CA USA; 3 Nuclear Threat Initiative, Washington, DC USA; 4 Department of Medicine, Stanford University School of Medicine, Stanford, CA USA; 5 Department of Microbiology & Immunology, Stanford University School of Medicine; and Center for International Security and Cooperation, Stanford University, Stanford, CA USA; 6 Schar School of Policy and Government, George Mason University, Washington, DC USA; 7 Center for the Study of Weapons of Mass Destruction, National Defense University, Washington, DC USA; 8 Center for Global Health Science and Security, Georgetown University, Washington, DC USA; 9 Center for Health Security, Johns Hopkins University, Baltimore, MD USA; 10 Media Laboratory, Massachusetts Institute of Technology, Cambridge, MA USA; 11 Department of Genetics, Harvard Medical School, Boston, MA USA; 12 The Johns Hopkins University Applied Physics Laboratory, Laurel, MA USA; 13 International Genetically Engineered Machine Competition, Boston, MA USA

**Keywords:** Synthetic biology, Policy

## Abstract

Biology can be misused, and the risk of this causing widespread harm increases in step with the rapid march of technological progress. A key security challenge involves attribution: determining, in the wake of a human-caused biological event, who was responsible. Recent scientific developments have demonstrated a capability for detecting whether an organism involved in such an event has been genetically modified and, if modified, to infer from its genetic sequence its likely lab of origin. We believe this technique could be developed into powerful forensic tools to aid the attribution of outbreaks caused by genetically engineered pathogens, and thus protect against the potential misuse of synthetic biology.

## Abstract (web summary)

A key security challenge with biosecurity threats is determining the responsible actor. In this Perspective, the authors review recent developments in using genetic sequence to assign a lab-of-origin and the potential protection it provides against misuse of synthetic biology.

## Introduction

Biotechnology is in an era of rapid and accelerating progress. Qualitative breakthroughs such as CRISPR and artificial gene drives unlock new capabilities, and quantitative trends show biotechnology as an area of increasing investment, decreasing costs, and expanding access.

However, alongside the benefits of this advancing technology for science, medicine, agriculture, and industry, there are concerns over its potential for accidental or deliberate misuse. Laboratory accidents have caused outbreaks before. The 2007 Foot and Mouth disease outbreak in the UK was attributed to a leaking pipe at Institute of Animal Health at Pirbright^1^. The last known cases of smallpox and SARS were both caused by laboratory exposures, and involved secondary transmission from infected researchers to individuals outside of the laboratory^2^. The 1977 influenza pandemic was caused by a strain closely related to those isolated in the 1950s, suggesting an anthropogenic origin^3^.

Both state and non-state actors have attempted to develop biological weapons in the last century. Although 183 states are party to the Biological Weapons Convention, which categorically bans the development and production of biological weapons, multiple states have been alleged to have violated the treaty. Non-state actors have also sought to use biological weapons^4,5^: notable among these are Al-Qaeda’s unsuccessful attempts to develop biological weapons^6^; Aum Shinrikyo’s ineffectual bioterrorist attacks^7^; and the Rajneeshee cult’s use of *Salmonella* to cause 751 cases of food poisoning in Oregon in 1984.

Technological progress magnifies these dangers: falling barriers to entry increases the risk that reckless or malicious actors will access biotechnology. Emerging capabilities may worsen the potential impact if this risk is realised. The 2011 ‘gain of function’ influenza experiments raised concern that adapting a highly virulent avian influenza strain to be transmissible between mammals posed an unacceptable risk since a laboratory escape could lead to a pandemic^8^. The increasing ease and accuracy of genetic engineering both widens the possibilities and lowers the barriers to entry to research that could be misused to produce pathogens more dangerous than naturally arising strains^9^.

## The attribution gap

Addressing these biological threats is an urgent and formidable challenge. One element of this challenge is attribution: being able to determine, in the wake of a human-caused biological event, who was responsible. Attribution has three main security benefits. First, knowledge of who was responsible can inform response efforts by shedding light on motives and capabilities, and so mitigate the event’s consequences. Second, it can identify the responsible parties for appropriate civil, criminal, or diplomatic penalty. Third, successful attribution followed by meaningful actions to hold perpetrators accountable can deter those inclined to reckless or malicious practice in the first place.

Information for attribution can be roughly divided into three categories. The first category includes non-technical indicators that provide contextual clues to intent, such as the victims, the location of the event, and epidemiological features. For example, if an incident occurs in the midst of an ongoing conflict, suspicion falls on the belligerents, while if it occurs near laboratories working on the causative agent, there is a greater chance of it being attributed to an accidental release.

Another category informing attribution is intelligence. This ranges from human sources, such as informers or whistleblowers; to intercepted communications; to surveillance data. All can potentially identify those responsible for the release of a biological agent.

The final category is technical forensics: the properties and characteristics of the agent that caused a given outbreak may provide clues as to who made it and/or who was responsible for releasing it.

The nascent field of microbial forensics helped the FBI identify a suspected lab of origin for the anthrax used in the 2001 attack and a suspected perpetrator responsible for the attack^10^. Nonetheless, further improvement of these forensic capabilities are a recognised need^11^. Two capabilities would be important: first, to establish whether the causative organism was genetically engineered; and second, if it was engineered, to identify the actor who engineered it.

To detect engineering, tools are being developed which can interrogate the genome of the causative organism for indicators of genetic engineering. The IARPA Finding Engineering-Linked Indicators project, FELIX, seeks to develop new experimental and computational tools for this purpose^12^. Under the auspices of the UN Secretary-General’s mechanism for investigating alleged biological attacks, there are separate efforts to develop an international trusted laboratory network that would provide forensic support to such investigations. As performance across laboratories in detecting genetic modifications is currently variable, the network may be strengthened through additional tools and access to existing technologies^13^.

Identification of the engineer poses a further challenge, since determining that an organism has been genetically engineered, and what that engineering involved, does not establish who the engineer was. A given set of edits could conceivably be performed by a multitude of different actors: from individuals working out of a community lab, to university research groups, to industrial laboratories, to a state-run bioweapons facility.

## Towards genetic engineering attribution

Fortunately, the very diversity of design approaches and technical options that are now available to achieve a given result (e.g. which genes or genetic features to use, their origin, and how to incorporate these genes or features into the genome) offers a means to approach the attribution problem. Which option a genetic engineer chooses will be influenced by a variety of factors, including their training, prior experience, habits, and available resources. In aggregate, these choices compose a ‘methodological signature’, and thus a way of tracing these design choices back to the likely designer.

That machine learning could be used to detect and interpret these signatures was demonstrated in late 2018^14^, although with a limited accuracy of 48%. Most recently, Alley and colleagues deployed deep learning techniques to predict lab-of-origin for plasmids submitted to the Addgene database - the largest repository of its kind, with 70,000 submissions from labs in 37 countries. Their approach offers an accuracy of 70% when distinguishing between over 1000 labs^15^.

They also pioneered further capabilities: uncertainty estimation, tracking ‘genealogies’ of genetic engineering groups, and inferring the nation in which the originating laboratory is located. Each of these has security promise: uncertainty estimation enhances robustness and can aid the integration of technical indicators with other available information for making an overall attribution decision; tracking lineages may identify other groups who knowingly or otherwise assisted the actor responsible; and the nation of the originating laboratory may provide a useful investigative clue in the absence of finer-grained information.

## The security potential of genetic engineering attribution

These rapid developments have potential as techniques, alongside publications and patents, to help understand patterns of influence and performance within the synthetic biology community, and also a means to identify and protect intellectual property. Our interest is in the biosecurity promise of using these advances to develop forensic tools which can aid attribution of genetically engineered agents and organisms.

The central benefit would be an increase in the actual and perceived accuracy of attribution decisions. This increases the likelihood of the right people being implicated in any misuse of genetic engineering in case of either an accident or an attack. The converse—avoiding mistaken attribution—is also key, given the potentially catastrophic consequences of one state mistakenly believing it is a victim of a biological attack.

An indirect effect of this improved accuracy is deterrence of misuse in the first place. Some actors may be incentivized to be reckless if they believe they are unlikely to be held accountable for any accidents arising from their actions. Malicious actors may be attracted to biological weapons as a means of clandestine violence. Better attribution tools deter both by increasing the risk of discovery.

Three additional features of genetic engineering forensics make it particularly attractive as a biodefense technology. First, unlike other instances where the interests of science and security conflict, the development of genetic engineering forensic tools does not impede scientific enquiry. If anything, it offers co-benefits for the overwhelming majority of well-intentioned and responsible genetic engineers: further means of receiving due credit and recognition, and further safeguards of their intellectual property.

Second, biodefense activity can paradoxically worsen security, by what is known as a security dilemma^16^. A given state’s biodefense activity, even if wholly defensive in intent, may nonetheless provoke concern in other states that this activity could both harbour and be co-opted for offensive purposes. Mutual suspicion can drive an arms race. Compared to other aspects of biodefense, genetic engineering forensics has more limited prospects for offensive use, and so state investment in this aspect of biodefense poses a lower risk of triggering suspicions and insecurity in its peers.

Third, the efficacy of genetic engineering attribution is coupled to biotechnological progress, so the trends that make misuse more concerning also enhance this approach to help address them. The rapidly growing corpus of genetically engineered sequence information provides more data that can be fed into these forensic tools; the increasing diversity of biotechnological methods also increases the diversity of ‘methodological signatures’ among practitioners.

## Challenges and next steps

The security benefits of genetic engineering attribution, even in the ideal case, would have limits. Attribution techniques are not techniques to detect whether engineering occurred in the first place: determining attribution is a process that would follow detection of engineering, and is not a substitute for it. Great caution should apply to using genetic engineering attribution as an improvised means of genetic engineering detection. Inability to attribute does not rule out genetic engineering: a sequence may show clear signs of engineering even if the engineer cannot be identified. There are also risks of false positives: improper use of genetic engineering attribution could ‘attribute’ non-existent engineering, such as identifying the ‘engineer’ of a wild-type pathogen genome.

Genetic engineering attribution is also not applicable to releases of non-engineered agents or organisms, for which other forensics methods remain necessary. Technical forensics may help identify the designer of the genetically engineered organism, but this may not be the actor who misuses it (although identifying the source of genetic engineering which is subsequently misused could be important information, for example in a case of suspected state-sponsored bioterrorism).

The deterrence value of attribution, and thus of better forensic tools to inform it, is sensitive to political context. Forensic identification offers little deterrence to actors intending to claim rather than conceal responsibility, nor to those who plan to evade the consequences of being held responsible by disinformation campaigns or other political means (although genetic engineering forensics may prove a harder target for disinformation if its techniques become public and well-characterised).

Realistic circumstances, rather than ideal ones, imply further limitations. 70% accuracy is far from a smoking gun, and although this may improve further, the performance ceiling is not known. Genetic engineering forensics should be seen as an important forensic tool in the attribution toolkit, instead of a standalone silver bullet.

A key uncertainty is that genetic engineering forensics has so far been developed on—and tested against—data from genetic engineers operating ‘in the clear’: those who publish their sequences to public repositories and make no attempt to conceal authorship. In the case of an attack rather than accident, sophisticated adversaries may also attempt to find ways of obfuscating or misdirecting attribution indicators—genetic engineering forensics included. For example, an attacker could attempt to adopt the ‘methodological signature’ associated with other practitioners in an attempt to deflect attribution or at least confuse the analysis.

Such attempts could leave their own trace, and forecasting how any potential contest between forensics and counter-forensics would play out is difficult; one side or the other may have an intrinsic advantage. Yet even in the worst case where an adversary is justifiably confident that they can evade genetic engineering forensics, doing so imposes a further cost, a further design constraint, and a residual risk of discovery. Each is a disincentive.

Genetic engineering forensics is at an early stage; there is a long way to go from published proof of principle studies to a robust forensic capability. These next steps include: First, starting a dialogue with the forensics and biodefense communities for what capabilities would be useful, and how technical forensic innovations can be brought into practice. Second, corralling further sources of data to improve accuracy and assess how performance scales. Third, leveraging ongoing improvements in machine learning and the creativity of practitioners to further improve the state of the art.

As biotechnology continues to pose a security challenge, it promises new tools to address the same. We believe it is the responsibility of the scientific and policy communities to identify opportunities to create these tools, like genetic engineering attribution, which reduce the risk of misuse. By engaging in this enterprise pro-actively, we can continue to realize the benefits of rapidly improving biotechnology while safeguarding biological security.
